# Towards safer use of pesticides in Chile

**DOI:** 10.1007/s11356-022-18843-6

**Published:** 2022-01-31

**Authors:** Jessica Coria, Sebastian Elgueta

**Affiliations:** 1 Department of Economics, University of Gothenburg, Gothenburg, Sweden; 2 Núcleo de Investigaciones Aplicadas en Ciencias Veterinarias Y Agronómicas, Universidad de Las Americas, Sede Providencia, Santiago, Chile

## Introduction

The use of pesticides for pest and disease control has increased agricultural production worldwide, but has also led to increased negative impacts on land use, the environment, and human exposure to various pesticide residues (Wilson and Tisdell 2001). Another important external effect is the unintended destruction of beneficial predators of pests, increasing the virulence of many types of agricultural pests. The economic costs of pesticide use have been greatly underestimated in the past. For example, Bourguet and Guillemaud (2016) evaluated the benefit–cost ratio of pesticide use in the past in different countries; their results suggest that the costs of pesticide use may have already exceeded its benefits. However, despite the high social costs, farmers continue to use pesticides, and in increasing amounts in most countries (Schreinemachers and Tipraqsa 2012).

Several studies have indicated that pesticide use has increased rapidly as countries prioritize overall food production over food safety and environmental concerns; as a result, pesticide regulations are often too weak to have a significant effect (see, for example, Lefebvre et al. 2015 and Grovermann et al. 2017). Yet, there are very few studies that look at the actual regulation of pesticides. This work contributes to such literature by analyzing the case of Chile, whose agricultural sector has experienced rapid export-led growth and progressive increase in the pesticide sector. Studying Chile’s experience allows us to discuss the challenges of controlling pesticide use, along with the rising demand for better controls. It is well acknowledged that the overuse of pesticide is a global problem. The analysis of the Chilean case brings light on the type of regulatory improvements that are needed towards a safer use of pesticides in many countries that, like Chile, are heavily dependent on the agricultural sector.

Our study is based on the analysis of secondary data and of existing national regulations. International regulations are also discussed as a benchmark for pesticide use. Finally, available literature on pesticide pollution in Chile and on strategies to reduce pesticide use are used to characterize the current situation and to draw recommendations on how to improve the effectiveness of current regulations.

Indeed, we focus on the case of Chile, a large producer of agricultural crops, because evidence points to a high rate of pesticide use in comparison with other countries. For instance, pesticide sales in OECD countries averaged 0.93 kg/ha in the 2011–2015 period. In contrast, pesticide sales per hectare in Chile corresponded to 2.68 kg/ha (see OECD/FAO 2019). The current pesticide registration in Chile covers more than 500 active ingredients and around 1300 formulations (SAG 2021).

Currently, the only regulation for the control of pesticide residues is related to food products, which is adopted from the International CODEX Alimentarius. In Chile, environmental matrices such as air, soil, water for irrigation, or human consumption are not regulated or monitored for pesticide residues levels. Nevertheless, empirical academic studies report that water bodies in agricultural areas present elevated levels of several pesticides (Retamal et al. 2013; Montory et al. 2017; Climent et al. 2018, 2019). For instance, high concentrations of organochloride pesticides have been found despite they have been banned in Chile in accordance with international conventions such as Rotterdam and Stockholm (e.g., endosulfan and lindane; see, e.g., Montory et al. 2017). Organochloride pesticides found in Chilean surface waters have been banned in developed countries since the mid-1970s due to their persistence and significant potential for bioaccumulation. The high concentrations observed in water bodies in agricultural areas in Chile can be explained by their high persistence in the environment, but it is also possible that they are still being used despite having been banned.

Furthermore, high concentrations of residues of pesticides such as methamidophos, azoxystrobin, cypermethrin, carbendazim, chlorpyrifos, cyfluthrin, and lambda-cyhalothrin have been found in leafy vegetables in some of the regions of the country (see, e.g., Elgueta et al. 2017, 2019, and 2020). In addition, high concentrations of residues of pesticides such as iprodione and spinosad A and D have been detected in frozen vegetables from domestic markets in Chile (see, e.g., Concha-Meyer et al. 2019). The widespread use of organophosphate pesticides in agriculture, their largely unrestricted sales, and insufficient knowledge of their proper application and risks have also resulted in acute intoxications (see, e.g., Ramírez-Santana et al. 2014) and chronic health effects in agricultural and non-agricultural workers, as well as in school children in rural areas (see, e.g., Muñoz-Quezada et al. 2012 and 2017). Evidence on the negative effects of pesticides thus suggests that there is a clear need for policies and strategies to reduce pesticide use and their impacts on human health and the environment.

The manuscript is organized as follows: “Pesticide use in Chile” describes the use of pesticides in Chile, presenting statistics on agricultural activity, imports, and the number of registered pesticides in use, and how many are banned or severely restricted in other countries. “Factors explaining high rates of pesticide use” discusses possible explanations for the high rates of pesticide use. “Conclusions” concludes with a discussion of strategies to reduce pesticide use and reduce the risk to the public and the environment.

## Pesticide use in Chile

Since the 1960s, Chilean agriculture has experienced rapid, increasingly diversified, export-led economic growth. The growth of the agricultural sector is evidenced by the increase in agricultural GDP, from 2.7% between 1963 and 1982 to 5.6% between 1983 and 2007, making it one of the pillars of the national export structure (INE 2007). The average value of the agricultural GDP in 2017 corresponded to 4.2%, slightly above the average value of the agricultural GDP worldwide, which is 3.5%. Even if as a percentage of national GDP, the primary agricultural sector might seem small, the extended agricultural GDP of agriculture increases to 14.36% when considering backward and forward linkages with other activities (ODEPA 2019).

Furthermore, agriculture accounts for 9.3% of employment, constituting one of the most important economic activities in terms of job creation, particularly in rural areas. Chilean Agriculture is characterized by a dual structure, where small-scale labor-intense farms coexist alongside a large-scale commercial farm sector. According to the last National Agricultural and Livestock Census (2007), more than 70% of farms are considered smaller than 20 ha, while over 20% range between 20 and 100 ha and 7.6% are larger than 100 ha (ODEPA 2019). Furthermore, family farms (which represent approximately 90% of the total farms) contribute a significant share of total farm output, particularly in horticultural products for domestic consumption (ODEPA 2019).

The agricultural sector makes an important contribution to exports, with agro-food exports accounting for 16.4% of total exports of the economy. Chile is a net exporter of agro-food products with a net trade surplus of USD 5 billion in 2017 (OECD-FAO 2019). The fruit sector is a major contributor to the development of exports; more than 60% of national fruit production in most species is exported.

**Fig. 1:**
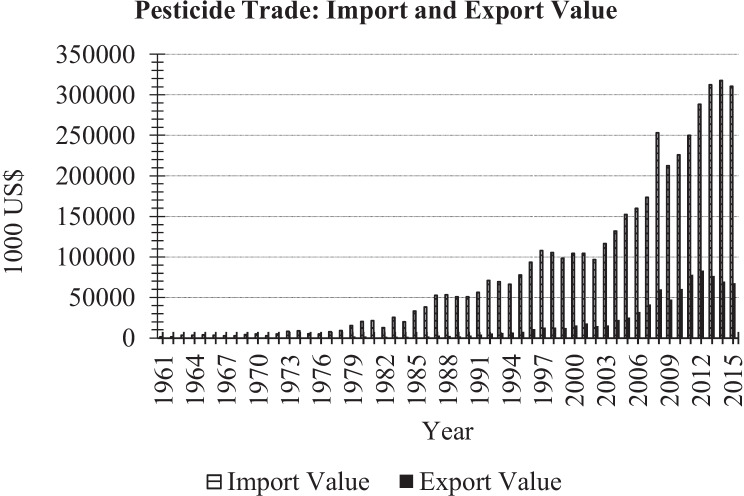
Total pesticide trade, import and export values 1960–2015. Source: FAOSTAT 2018

The “boom” in Chilean agriculture has, however, caused a progressive growth of imports of pesticides, which rose by 48% in the decade of the 1990s (Vallebuona Stagno 2015). This increase in pesticide use occurred in an unregulated environment of free sale and easy access, accompanied by a lack of knowledge of pesticide use and their impact on human health and the environment (Vallebuona Stagno 2015). Figure 1 presents the total pesticide trade from 1960 to 2015. The total imports of pesticide by Chile amounted to US$ 310,515 million in 2015, almost three times larger than the values of the imports in 2000, suggesting a very rapid growth in the intensity of pesticide use.

**Fig. 2:**
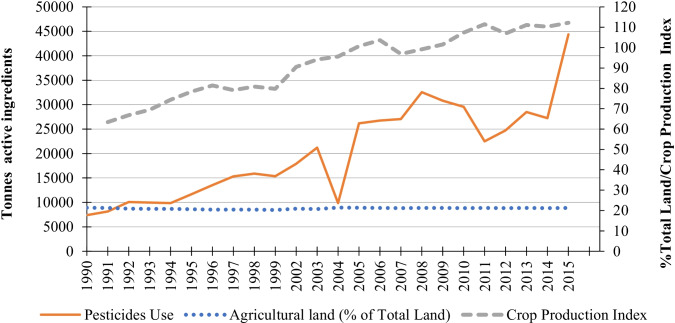
Pesticide use vs. crop production and agricultural land 1990–2016. Source: FAOSTAT 2018

Indeed, as shown in Fig. 2, pesticide use (measured by tones of active ingredients) and agricultural production (measured by a crop production index that reports the level of annual production compared to the period 2004–2006) have increased sharply and steadily (i.e., in 2015, six times more pesticide was used than in 1990) while the share of total land devoted to agricultural activities has remained quite stable during the same period (i.e., about 21% of the total land is used in agricultural activities). This is to say, pesticide use increased more than proportionally to land use intensity; regressing pesticide use on the crop production index suggests that a 1.0% increase in the crop production index is associated with a 2.37% increase in pesticide use (*p*-value < 0.00 and *R*^2^ = 0.76).

Sales of pesticides are concentrated in the central part of the country due to the intense farming and forestry activity that takes place between the Chacabuco Range in the north and the Biobío River in the south. For instance, out of 38,864 tones/L of pesticides sold in Chile in 2012, 30,643 tones/L (about 78.8%) were sold in the central area of Chile (SAG 2012).

Until December 2020, there were more than 1300 plan protection products (PPPs) authorized in Chile, classified as Series 1000 (insecticide), 2000 (fungicide), 3000 (herbicide), and 4000 (other), containing more than 580 active substances (SAG 2021). Out of the total authorized products, 29.7% are locally manufactured while the rest are imported (Economic Diplomacy Division, MEA 2018). Several of the authorized substances cannot be legally used in developed countries. This is the case for paraquat, carbofuran, permethrin, brodifacoum, tetramethrin, and fenitrothion, which have been banned due to their hazardous effects on human health and the environment (see Table 1). Moreover, there are around 28 active substances of pesticides prohibited for use in Chile. The last ones were forbidden in 2011 (i.e., pentachlorobenzene, endosulfan, and alachlor) and in July 2019 (i.e., methamidophos and chlorpyrifos).

**Table 1:** Known effects of pesticides authorized in Chile and prohibited in other countries

Pesticide	Type	Countries where pesticide is banned	Known health and environmental impacts
Paraquat	Herbicide	EU, South Korea	Parkinson’s Disease (Spivey 2011), Suicides (Cha et al. 2016)
Carbofuran	Insecticide	EU, Canada	Potential Endocrine Disruptor (Goad et al. 2004)
Permethrin	Insecticide	EU	Neurotoxic effects (Abou-Donia 1996), toxic to honey bees, fish, aquatic insects, crayfish, and shrimp (Brander et al. 2016)
Brodifacoum	Insecticide	EU	Lethal to birds (Hoare and Hare 2006)
Tetramethrin	Insecticide	EU	Causes restlessness, hyperexcitation, prostration, body tremors, and repetitive nerve discharges (Palmquist et al. 2012)
Fenitrothion	Insecticide	EU	Toxic to aquatic organims, extremly toxic to honey bee and non-target arthropods (Calatayud-Vermich 2018)

As shown in Table 2, in 2018, most imports of insecticides, fungicides, and herbicides (which correspond to the main imports’ categories) came from Argentina, USA, China, and Brazil. As shown in the table, in general, the volume imported from a country is inversely correlated to the average price of the products. In the case of insecticides, products imported from Argentina and China are the cheapest among all insecticides imported. Likewise, fungicides from the USA and China are among the cheapest of all fungicides imported, and the same holds for herbicides (of which large amounts are imported from Argentina and China).

**Table 2:** Imports of pesticides by country in 2018

Country	Insecticides		Fungicides		Herbicides	
	kg	US/kg	kg	US/kg	kg	US/kg
Argentina	4,111,602	2.2	43,666	28.7	4,325,860	3.9
USA	488,987	27.1	3,263,391	4.9	464,648	16.2
China	1,521,650	4.3	523,486	2.4	2,313,312	3.6
Brazil	128,350	18.0	312,682	16.1	1,184,450	5.0
France	37,705	59.0	357,803	24.3	620,127	16.7
Israel	179,782	6.8	420,708	9.9	475,326	6.2
Germany	142,481	62.3	218,053	28.2	456,268	25.1
Colombia	381,611	12.1	455,348	25.4	179,156	18.2
UK	33,314	145.3	95,675	50.0	432,202	3.2
Spain	16,766	47.9	479,336	7.3	–	–
India	35,135	6.5	371,023	3.0	–	–
Japan	154,194	84.9	87,072	17.9	14,142	69.5
Belgium	2016	50.2	209,175	10.6	44,307	5.7

Source: Elaborated from data provided by ODEPA (Office of Agricultural Studies and Policies)

Thus, the evidence presented in Table 2 is consistent with a large volume of imports of low-cost products from a few countries, including Argentina, the USA, and China. This is confirmed by evidence provided in Table 3, which reports the country of origin of selected imported pesticides for which high concentrations have been found in water adjacent to agricultural land or in agricultural products.

**Table 3:** Country of origin of selected pesticides

Pesticide	Countries from which pesticide is imported
Paraquat	Brazil, Chile, China, UK
Carbofuran	Brazil, USA
Permethrin	Brazil, USA, China
Brodifacoum	Brazil, Chile, Hungary
Methamidophos	Argentina, Chile, China, USA

Source: Elaborated from the SAG registry of pesticides authorized in Chile

### Pesticides prohibited and presence of residues in food

Legislation on the marketing and use of PPPs is laid down by Decree Law No. 3557 of 1980. The resolutions for the implementation of this law include requirements for the authorizations of PPPs and obligations for retailers of PPPs to report their sales, as well as any stocks of products with expired shelf life (European Commission 2009).

Regarding the presence of residues in food and the environment, in Chile, the pesticide maximum residue levels (MRLs) are adopted from Codex Alimentarius, based on the USA and Europe, and were established by Resolution No. 581 of 1999 of the Ministry of Health, later replaced and updated by Resolutions 33/10 and 762 of 2011 of the Ministry of Health.

Article 34 of Decree Law No. 3557 established that the Agriculture and Livestock Service (SAG)—under the Ministry of Agriculture—is the competent authority for authorization, importation, manufacture, distribution, sale, and use of PPPs and control of their marketing and use, and that SAG can confiscate or forbid the movement and sale of fruit and vegetables containing pesticide residues above the legal limits or pesticides not authorized for the crops. In contrast, the Ministry of Health is the competent authority, through the National Institute of Public Health (ISP), for checking compliance with MRLs for fruits and vegetables sold on the domestic market, including retailers and local street markets. Each ministry has its own surveillance system to evaluate the agricultural use of pesticides and the impacts of pesticide residues on human health. Thus, there is no unified national surveillance system.

**Fig. 3:**
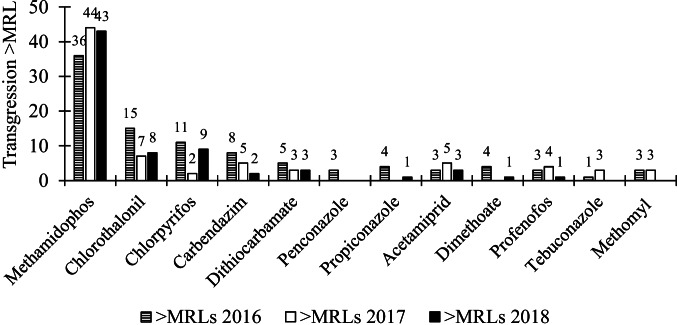
Notifications in Chile by pesticide related to their maximum residue level (MRLs) and pesticide prohibited for fresh vegetables. Source: RIAL (2018, 2019, 2020)

The Ministry of Agriculture monitors pesticide residues in food through the Chilean Agency for Safety and Food Quality (ACHIPIA). This agency is in charge of the Information Network and Food Alerts program (RIAL), which started in 2011. The last official RIAL report published in 2020 presented the results of 1306 samples of fresh vegetables (407) and fruits (899) taken in 2018. The sampling is carried out across all of Chile by inspectors from SAG. Among the samples of vegetables evaluated, almost 19% of the vegetables exceeded the MRLs, while almost 9% contained unauthorized pesticides (see Table 4). A low level of compliance was also observed in the report published in 2019 (with samples taken in 2017), where almost 17% of the vegetables exceeded the MRLs, and almost 7% contained unauthorized pesticides. Slightly higher levels of compliance were observed in the samples of fruit taken in 2016. Among the evaluated samples of fresh vegetables, the highest MRLs exceedance rate was identified for methamidophos, linuron, chlorothalonil, acetamiprid, and dithiocarbamate (see Fig. 3).

**Table 4:** Results of sampling of vegetables and fruits under the RIAL program 2016, 2017, and 2018

Year	Total sample vegetables	Total sample fruits	Fresh vegetables		Fresh fruits	
			% samples > MRL	% samples with unauthorized pesticides	% samples > MRL	% samples with unauthorized pesticides
2018	407	899	18.6%	8.6%	1.20%	2.70%
2017	485	1103	16.9%	6.8%	1.09%	3.45%
2016	781	1175	13.7%	4.1%	0.77%	2.21%

Source: RIAL (2018, 2019, 2020)

More than 60% of fresh vegetables consumed in Chile are distributed through local street markets, which may explain the lack of food safety enforcement. Data reported by the RIAL program show a recurrent presence of pesticide residues in fresh vegetables, suggesting that Chilean consumers are regularly exposed to the acute and chronic toxic effects of pesticides such as methamidophos, chlorothalonil, and methomyl through food consumption. Evidence presented by RIAL (2018, 2019, and 2020) is also consistent with studies produced by the Chilean Institute for Agricultural Research (INIA). For example, Elgueta et al. (2017) quantified the concentration of pesticide residues in leafy vegetables in 118 samples of lettuce, chard, and spinach collected in 2015 in three north-central regions of Chile. Their results showed that 27% of the total samples contained residues above the MRLs. Their study also concluded that methamidophos is the pesticide with the greatest acute health risk to the population. In addition, Elgueta et al. (2019) collected 53 samples of leafy vegetables from markets in Santiago, Chile, to assess the health risk due to pesticide residues. Pesticides such as carbendazim, chlorpyrifos, cyfluthrin, lambda-cyhalothrin, and methamidophos were frequently detected in their samples. Of the total samples evaluated, 11% had residues above the Chilean MRLs. Similar results were also found for fresh tomatoes and lettuce grown in the Metropolitana Region (Elgueta et al. 2020), where 16% of 80 samples were found to have pesticide residues above Chilean MRLs. For comparison, the annual reports available at the European Food Safety Authority show that exceedance of MRLs in Europe in 2013 only occurred in 2.6% of the cases (EFSA 2015). Existing evidence thus indicates a much higher risk for the Chilean population compared to Europe.

In contrast, Chile’s system for monitoring pesticide residues in agricultural exports shows a very high rate of compliance. For example, in 2009–2010, SAG took 1639 samples of vegetables to be exported. Of these samples, 99% complied with MRLs and used only approved pesticides. Similar numbers were observed in 2011 for exported vegetables and in 2012 for exported fruits (see SAG 2011, SAG 2012, and SAG 2013). The fact that high levels of pesticide residues are found mainly in products intended to be consumed domestically indicates a lack of monitoring and enforcement within Chile.

### Occupational, non-occupational, and environmental effects of pesticides

Regulation of occupational exposure to pesticides in Chile depends on the Ministry of Health with the support of the Institute of Public Health. Law 16.674 and its implementing regulations, enacted in 2004, created a protocol for national notification of acute pesticide poisoning reports. In addition, Regulation D.S 594/2001, which establishes health and environmental conditions for workers, regulates the aerial application of pesticides in urban areas and the monitoring of pesticide use by agricultural workers.

Statistics collected by the System of Monitoring of Acute Pesticide Poisonings (REVEP) indicate that a total of 3914 cases were reported from 2012 to 2017, with an annual average of 652 cases and a cumulative incidence rate of 3.6 per 100,000 inhabitants. The cases of acute pesticide poisoning are divided into occupational, accidental (not labor), and suicide attempts. Occupational poisoning (41.5%) and accidental non-work poisoning (41.7%) constitute the largest number of cases, totalling more than 83%. Suicide and suicide attempts make up around 17% of the total cases (REVEP 2019).

Table 5 reports the number of cases of acute pesticide poisonings by active ingredient. As shown in the table, the use of cypermethrin, chlorpyrifos, lamba-cyhalotrin, methamidophos, diazinon, alpha-cypermethrin, glyphosate, azinphosmethyl, brifacoum, deltamethrin, methomyl, and paraquat account for almost 50% of all acute pesticide poisonings in the period 2008–2018 in Chile. Considering all statistics during this period, 35.5% of deaths were caused by pesticides Ia and Ib (REVEP 2019). These insecticides are highly efficient, low cost, and highly toxic, which explains the incidence of poisoning among Chilean agricultural workers. In 2018, the main pesticides related to acute pesticide poisoning were lamba-cyhalotrin (17.5%), chlorpyrifos (17.5%), cypermethrin (5.2%), and methamidophos (5.1%) (REVEP 2019).

**Table 5:** Acute pesticide poisonings by active ingredient 2012–2017

Active ingredient	2012	2013	2014	2015	2016	2017
Cypermethrin	87	79	132	46	78	51
Chlorpyrifos	106	38	21	140	5	30
Cyhalothrin	42	50	136	20	7	48
Glyphosate	19	46	19	15	17	33
Bromadiolone	5	19	6	8	8	100
Methamidophos	22	25	42	21	12	22
Alpha-cypermethrin	11	0	12	4	45	69
Azinphos methyl	118	3	14	1	0	1
Diazinon	30	11	52	4	6	11
Brodifacoum	15	12	12	15	9	21
Hydrogenated cyanamide	16	22	11	9	14	9
Methomyl	0	18	13	9	19	18
Deltamethrin	0	5	11	42	0	8
Sulfur	32	8	10	9	3	2
Others	370	206	337	240	215	227
Total	873	542	828	583	438	650

Source: REVEP (2018)

A recent study by Boedeker et al. (2020) estimates the global distribution of acute unintentional pesticide poisoning based on a systematic review supplemented by mortality data from the World Health Organization database. They estimate that about 385 million cases of acute unintentional pesticide poisoning occur annually worldwide including around 11,000 fatalities. Given a worldwide farming population of approximately 860 million, they estimate that about 44% of farmers are poisoned by pesticides every year. The figures for Chile indicate that annually 17.63% of the national farmers are poisoned by pesticides, well below the median values for South America and the world. Chile has made progress in adopting better pesticide application practices and monitoring exposed workers, and acute pesticide poisoning is not the main hazard associated with the high use of pesticides in the country. On the other hand, the actual incidence of pesticide poisoning could be much higher than official records because workers do not inform the authorities due to ignorance or fear of losing their jobs (see, for example, Ramírez-Santana et al. 2020).

Furthermore, the current surveillance system focuses on acute pesticide poisonings, while little is known about the chronic effects of exposure to pesticides through agricultural applications or consumption of contaminated food by the general population. In fact, very few studies in Chile have examined non-occupational exposure to pesticides and their effects. These few studies have found high chronic exposure to organophosphate pesticides (OP) in agricultural and non-agricultural workers, as well as in school children in rural areas (see, e.g., Muñoz-Quezada et al. 2012 and 2017). In addition to acute poisonings in children or adolescents (which account for one-third of reported non-occupational cases, see Ramírez-Santana et al. 2020), studies have shown that children have cumulative exposure to OP insecticides, pyrethroid pesticides, and herbicides through their diet, residential use, living near farms, and through parents who work in agriculture (see, e.g., Muñoz-Quezada et al. 2012, 2017, 2019, and 2020). Studies have also linked pesticide use to spontaneous abortions, births of children with congenital malformations and reproductive health changes, cytogenetic damage in farm workers, decreased cognitive performance in the general population, and neurotoxic effects that may reduce cognitive performance in developing children and degenerative diseases in chronically exposed farm workers (see, for example. e.g., Szot 2004, Zuñiga et al. 2007; Lucero et al. 2019, and Ramírez-Santana et al. 2020).

Although there are few studies related to water contamination by pesticides in Chile, the research carried out to date consistently reveals the presence of agrochemicals in the surface water of agricultural areas of the Central Valley of Chile. For instance, Climent et al. (2019) found the presence of 22 pesticides and 12 degradation products in the Cachapoal River basin; Montory et al. (2017) found organochlorine compounds in surface water of the Ňuble River basin; Giordano et al. (2011) found residues of diazinon, lindane, chlorpyrifos, cyhalothrin, cypermethrin, and fenvalerate in the Itata River; Palma et al. (2004) detected simazine, hexazinone, 2,4-D, picloram, and carbendazim in the water of the Traiguén River; Cooman et al. (2005) reported the presence of atrazine residues in the Chillán River; while Dutka et al. (1996) detected triazine, atrazine, metolachlor, and benomyl in the surface water and sediment in Temuco and Rapel River basin. All of these were reported at levels exceeding the European regulations (0.1 µg ^L−1^), except in Dutka et al. (1996), who detected lower pesticide concentrations. Pesticides discharged directly into the river, washing of empty pesticide containers, pesticide overuse, and chemical fallow, have been identified as the main sources accounting for the presence of organochlorine pesticides (Montory et al. 2017). Thus, the existing evidence demonstrates the need for surveillance system to monitor pesticides in surface water areas of Chile and propose different corrective measures to reduce pesticide contamination. At present, Chile lacks a surveillance system of pesticide residues in water and soil, especially in agricultural areas. Therefore, policies should be developed and enforced to prevent environmental exposure of the general population around farming areas through water contamination.

## Factors explaining high rates of pesticide use

An obvious question is why the rate of use of pesticides in Chile is as high as it is. Potential explanations are the intensification of the agricultural activities in the country, the lack of information related to the potential impacts of pesticides on human health and environment, lack of monitoring and enforcement of current regulations, and the low cost of pesticide products. In what follows, we discuss each of these drivers.

### Intensification of agricultural activities

As discussed previously, the boom of agricultural production has caused a progressive increase in the use of pesticides. While the use of pesticides has resulted in large yield increases and improved agricultural productivity, the negative health and environmental effects of pesticide use raise concerns regarding their sustainability. Pesticide overuse results from a market failure as agricultural producers fail to internalize the negative externalities of pesticide overuse. Even government regulators in the most advanced industrialized countries have been unable to fully correct such market failures, as evidenced by the fact that few countries have reduced pesticide use (see, e.g., Lefebvre et al 2015). Reducing pesticide dependence requires a shift to integrated pest management that harmonizes biological and chemical controls instead of fully relying on chemical pesticides. Empirical evidence has shown that the application of such an approach can lead to substantial reductions in pesticide use without leading to a decline in crop yields. For example, Pretty and Bharucha (2015) provide evidence of the impacts of integrated pest management on productivity and pesticide reliance in a sample of countries in Africa and Asia. They find the reduced reliance on pesticide does not lead to reductions in productivity. In contrast, it can lead to a wide range of on-farm and off-farm benefits, such as savings, improved public health, and natural capital and around farms.

### Lack of information related to the potential effects of pesticide use on human health and environment

It has been argued that farmers’ intentions to secure crops through over-spraying and the lack of pesticide enforcement are the main factors contributing to the overuse of pesticides (see, e.g., Marcoux and Urpelainen 2011). Farmer education, gender, and limited access to technical assistance, as well as pesticide risk perceptions, influence pesticide overuse (see, e.g., Matthews 2008 and Liu and Huang 2013). Regarding risk perception, Dreyer et al. (2016) examined how different socioeconomic variables influence smallholder farmers’ risk perception of pesticides in Chile. According to their study, smallholders perceive the actual risk of pesticides to be low because there is little and uncertain evidence of visible health damage. As previously described, a relevant fact that neutralizes the concern about pesticides is the economic need to produce and work; even if the use of pesticides might pose some risks to their health, it appears as a necessary and comparatively minor evil.

Farmers prefer to use chemical pesticides because of their efficacy, without understanding how reliance on pesticides can lead to a loss of natural pest enemies, the development of pesticide resistance, and the resulting need to use pesticides more frequently in the future (Dreyer et al. 2016). In addition, there is a coordination problem: because farmers’ land is surrounded by plantations that apply pesticides, they believe that if they do not apply pesticides, they will be more vulnerable to infestation by the pests. Thus, even if farmers believe that organic farming or integrated pest management is more beneficial for environmental sustainability and less risky for health, they perceive several difficulties associated with this type of organic farming. The most important is the greater risk of not being able to control the pest, resulting in losses or damage to their production. It also means more work and therefore increased production costs.

Increased awareness about the risks related to pesticide exposure has shown not to be associated with lower exposures by Chilean farmers. This is because factors beyond the control of individuals, such as nearby agricultural use and residues in food, are the primary determinants of exposure (see, e.g., Muñoz-Quezada et al. 2019). Therefore, it is necessary the implementation of enforcement and control of pesticides with regulation from government institutions. One of the aspects that strongly influences farmers’ practices is the market. If this would require low levels of toxicity, producers would be concerned with compliance. Therefore, incentives and regulations that induce reorientation of the market could have positive effects on preventive practices. Under the current situation, when MRLs are violated, supermarkets and food processing companies will not purchase these products. In contrast, MRLs of products sold in local street markets in Chile are not verified. Hence, fruits and vegetables exceeding MRLs can still be sold. This is to say, the lack of enforcement of pesticide residues in fresh food sold in local street markets entails a major risk for consumers, who run the risk of purchasing products that contain high concentrations of pesticide residues over the MRLs or pesticides prohibited for the crop.

Furthermore, the amount of money Chilean farmers spend on pesticides is higher than necessary because of the lack of training and knowledge. Crop insurance can contribute to reduce the use of pesticide in agriculture by providing a substitute for the risk management benefits of pesticides. Empirical studies worldwide have shown that crop insurance leads to more environmentally friendly behavior from farmers (see, e.g., Feinerman et al. 1992 and Aubert and Enjolras 2018). In Chile, there has been a crop insurance program in place since the year 2000 with the aim to protect farmers against climatic events. The insurance cover fruits, vegetables, and annual crops with a policy to protect farmer of up to two-thirds of the potential value of the crop (Salazar et al. 2019). However, the use of insurance in Chile is quite low, even if the price of the insurance is partially subsidized. For instance, in 2014, only 6.4% of the total farm area was insured (see, e.g., Salazar et al. 2019). Lack of knowledge about the existence and potential benefits of crop insurance seems to be the main reason for the low uptake. A potential avenue of action for the Chilean government to reduce the use of pesticides could then be to promote the uptake of crop insurance and to tie the magnitude of the subsidies to the sustainability of the agricultural practices.

### Monitoring and enforcement of pesticide regulations

Regarding the monitoring and enforcement of current regulations, the evidence presented suggests a lack of appropriate control of pesticide regulations, as well as a lack of a modern pesticides’ approval/registration procedure. In particular, to reach the standards of developed countries, stricter requirements to purchase pesticides, application records, and training should be implemented, such that traceability of pesticide use is possible. Moreover, for the control of pesticides sales in the local market, a certification should be implemented by the authorities to identify all the stockholders involved in the value chain of pesticides. The registration process, storage, commercialization, and final customer should also be identified.

Furthermore, the Agricultural and Livestock Services, SAG, should improve the availability of information on pesticide sales per year, which currently is available with a huge temporal lag between the year in which the sales take place and the year in which such data is publicly available. Such information would allow conducting evaluations and making decisions regarding public policies to control pesticide use.

Several countries require written “recipes” by an accredited advisor to sell pesticides. Such recipes specify the type of product and the quantity, at least for those that have a higher level of toxicity. This is an aspect where regulation needs to be improved in Chile where pesticides can be bought without a justified technical recommendation. Anyone in the country can buy pesticide with active ingredients Ia and Ib considered highly toxic by the World Health Organization.

### Low cost of pesticides

Even though government support for Chilean agriculture creates limited distortions in agricultural markets because there are almost no price subsidies to farmers, the hidden costs and externalities of pesticide use are not taken into account in policy decisions. In order to reduce pesticide use, the high external costs of pesticides need to be considered in policy decisions and restrictions placed on those hazardous pesticides that cannot be safely managed (McCann 2005). However, in Chile, as in many other countries, policy makers are very reluctant to restrict pesticide use for fear of affecting food production, reducing export revenues, or increasing food prices, although these fears are often not based on an empirical analysis of the associated costs and benefits. In the absence of such analysis, policy debates on pesticide issues are vulnerable to the influence of ideology and commercial interests.

There is increasing debate in developed countries about the use of taxes and other regulatory measures to reduce pesticide use. For example, France aims to reduce agrochemical use by 50% by 2025, with measures that include bonuses and penalties for those who do not meet the target (similar to what some countries have introduced for energy savings). However, the demand for pesticides is quite inelastic (see, e.g., Böcker and Finger 2017) possibly due to the increasing importance of pesticides in current agricultural production practices compared to previous decades (see, e.g., Skevas et al. 2012). Since the demand for pesticides is quite inelastic—and even more inelastic for more toxic pesticides (see, e.g., Skevas et al. 2012)—large amounts of taxation may be needed to achieve substantial volume reductions.

In many developed countries, requirements to reduce pesticide use arise from consumer demand (rather than regulatory requirements). Consumers are increasingly asking questions about the food they consume and how it is produced. This has led to an increasing demand for organic products around the world. In Europe, for example, trade in organic products has increased significantly in recent years. In contrast, the Chilean domestic market for organic products is still in its infancy (see Padilla Bravo et al. 2012). The main obstacle to the development of a domestic organic market is the lack of consumer knowledge about the characteristics of organic products and the potential benefits of organic farming, together with the high price premiums for organic products compared to conventionally grown alternatives. As a result, commercial organic production in Chile is export-oriented and thus subject to increasing demands for organic food standards from foreign customers.

Traceability is one of the most important measures to ensure the safety and quality characteristics of food. In countries such as the USA, traceability systems are usually motivated by economic incentives rather than government regulations on traceability (see, e.g., Golan et al. 2004). Companies build traceability systems to improve supply-side management, increase safety and quality control, and market food products with credibility attributes. In markets where final or intermediate demand is strong enough to cover the cost of product differentiation, manufacturers have responded with new products and new traceability systems to support credibility attribute claims, including food safety claims. To eliminate potential fraud or unfair competition, industry groups and individual companies are increasingly relying on the services of external auditors to verify the existence of credibility attributes.

## Conclusions

Chile produces and exports more food than ever before. At the same time, concerns about the quality and safety of products consumed in the country have increased due to reports showing poor use of pesticides. In addition, pesticide exposure affects the health not only of farm workers but also of people from the general population. Environmental exposure to organophosphate pesticides occurs, for example, through pesticide residues in fruits and vegetables, spraying near farmland, and pesticide residues in water and soil. Improvements in the regulation and control of pesticide use are clearly needed.

The overuse and misuse of pesticides in agriculture often begin with consumers being largely unaware of their exposure to pesticides and the potential risks associated with them, and farmers lacking knowledge and training on the necessary safety measures (Xu et al. 2008). Raising awareness through training and education is crucial as it promotes farmers’ interest in alternative methods of crop protection, creates political support for the implementation of necessary policies, and influences consumers to demand safe food (see, e.g., Schreinemachers and Tipraqsa 2012). It is also important to improve farmers’ understanding of the ecology of pest management, such as the unintended negative effects of pesticides on natural predators of pests. When farmers are well educated on the use of pesticides and their effects through good agricultural practices, they are less likely to violate maximum residue level regulations and consumers can trust the quality and safety of food (Damalas and Koutroubas 2017).

Outreach can increase public awareness about the risks associated with the overuse and misuse of pesticide, which can help reduce pesticide use in the long run. However, stricter regulations on the sale and use of pesticides and improvements in monitoring are also needed. The Ministry of Agriculture, through the Agricultural and Livestock Service (SAG), regulates matters related to the import and sale of pesticides for agricultural use. However, the last publicly available record of sales is from 2012, and the lack of current information makes it difficult to determine with certainty the extent of occupational and environmental exposure to pesticides and the adverse public health effects. In addition, there is concern that highly hazardous pesticides are being used with inadequate control. Based on the current data published by RIAL and REVEP, decision-makers should remove from the Chilean market some highly hazardous pesticides with MRLs above the standards. Pesticides such as cypermethrin and methomyl should be banned from the Chilean market due to their hazardous effects, high levels in fresh food, and exceeding MRLs. In addition, Chile should advance pesticide traceability through stricter requirements for purchasing products and by implementing a system for documenting applications for pesticide sales and use.

Coordination and collaboration between Chile’s agricultural agency Livestock Service and the Ministry of Health are critical to the effectiveness of monitoring, compliance, and enforcement related to pesticides and their effects on human health and the environment. Ensuring an adequate level of protection requires effective regulatory oversight, up-to-date legislation, and regular evaluations to ensure that national laws are adopted, enforced, and effective. There is a need to create monitoring systems to trace pesticide residues in air, soil, and water for irrigation and human consumption. There is also a need to improve national capacity and develop practical tools to monitor and regulate pesticide trade. In addition, the list of registered pesticides should be regularly reviewed and updated, with the aim of reducing the use of high-risk pesticides and promoting the use of environmentally friendly alternatives in agriculture.

Chile is heavily dependent on agriculture; therefore, it is important to improve agricultural management to ensure its sustainability. Improved agricultural practices can not only reduce the negative health and environmental impacts of agriculture, but also open access to new, more profitable markets that require high levels of food safety and pesticide traceability.

## Data Availability

Data sharing is not applicable to this article as no datasets were generated or analyzed during the current study.
